# Modelling π-electron energy of benzenes via Zagreb psi indices

**DOI:** 10.1007/s10822-026-00797-3

**Published:** 2026-04-10

**Authors:** Kerem Yamaç

**Affiliations:** Van Yüzüncü Yıl University, Van, Turkey

**Keywords:** QSPR studies, π-electron energy levels, Benzenes, Topological indices, Psi degree, Zagreb psi indices

## Abstract

The new third Zagreb psi index achieves a correlation coefficient of 0.99948 for modeling the π-electron energy of benzenoid hydrocarbons, outperforming all previously reported degree-based topological indices. In this study, a novel vertex descriptor, referred to as the psi degree, is introduced by combining additive and multiplicative contributions of neighboring vertex degrees in a unified manner. Based on this concept, three Zagreb-type psi indices are defined and systematically analyzed within a quantitative structure–property relationship (QSPR) framework. Correlation and linear regression analyses demonstrate the strong predictive ability of the proposed indices, with particular emphasis on the third Zagreb psi index. In addition, structural sensitivity and smoothness analyses are performed to examine the behavior of the new descriptors under local structural modifications. While the present investigation is restricted to benzenoid hydrocarbons and linear modeling, the results indicate that Zagreb psi indices constitute chemically meaningful and promising tools for QSPR studies, motivating further applications to other molecular classes and physicochemical properties.

## Introduction

Topological indices provide numerical descriptors of molecular graphs that encode structural information and enable the quantitative modeling of physicochemical properties of chemical compounds. Since the pioneering works of Wiener and Platt, which established the relationship between molecular structure and boiling points of hydrocarbons, graph-theoretical descriptors have played a central role in quantitative structure–property relationship (QSPR) studies [1, 2]. Subsequent developments, including the Zagreb indices and the Randić connectivity index, further demonstrated that degree-based descriptors can effectively capture essential structural features of molecules and correlate strongly with experimentally measured properties [4, 5].

Over the past decades, a vast number of topological indices have been introduced, many of which are based on vertex degrees or combinations thereof [6–8]. Classical and extended Zagreb-type indices, atom–bond connectivity indices, and arithmetic–geometric indices have been successfully applied to model various physicochemical properties of organic compounds and nanostructures [10–16]. More recently, renewed interest in degree-based descriptors has led to the proposal of new families of indices, such as the Sombor and Nirmala indices, which have shown notable predictive performance in QSPR applications [17–20].

Benzenoid hydrocarbons occupy a particularly important position in chemical graph theory and QSPR research, as their highly regular structure and pronounced π-electron delocalization make them an ideal testbed for evaluating the chemical applicability of newly defined molecular descriptors. In this context, several studies have focused on modeling the π-electron energy of benzenoid hydrocarbons using both degree-based and distance-based topological indices [21–23]. Distance-spectral descriptors and recently proposed degree-based indices, including the Elliptic Sombor index, have demonstrated remarkably high correlations with π-electron energy, highlighting the sensitivity of this property to subtle structural variations in benzenoid systems [22–24].

Parallel to these developments, the classical Zagreb framework has been actively extended through the introduction of new degree-based variants aimed at improving QSPR performance. Recent studies have proposed Zagreb rho, upsilon, eta, and omicron indices and demonstrated their effectiveness in modeling physicochemical characteristics of benzene and benzene derivatives [30–33]. Although these indices enrich the Zagreb family and improve predictive accuracy in certain cases, they are still fundamentally constructed from single-degree contributions or simple edge-level degree combinations. As a result, the local neighborhood structure of a vertex is typically represented in a limited additive manner.

Despite the abundance of degree-based topological indices in the literature, the issue of structural sensitivity and smoothness remains a critical criterion for assessing their chemical applicability [3, 26–28]. In particular, the extent to which a descriptor simultaneously reflects additive and multiplicative interactions among neighboring vertices has not been systematically explored in the context of π-electron energy modeling. To the best of our knowledge, no previously proposed Zagreb-type descriptor explicitly integrates both the sum and the product of neighboring vertex degrees into a unified vertex-level quantity and examines its relevance for benzenoid hydrocarbons. Related efforts focusing on neighborhood-based and exponential structure descriptors have been reported in recent years for benzenoid hydrocarbons and closely related graph classes, further emphasizing the importance of local structural information in QSPR modeling [34, 35].

From a chemical perspective, the motivation behind the psi degree lies in the fact that π-electron delocalization in benzenoid hydrocarbons is influenced not only by the number of adjacent bonds but also by the collective interaction of neighboring atomic environments. The additive component reflects local connectivity, whereas the multiplicative component emphasizes cooperative neighborhood effects that cannot be captured by purely additive degree measures. The application of the nth root serves as a normalization mechanism, ensuring scale balance and allowing meaningful comparison across molecular graphs of different sizes.

Motivated by this gap, the present work introduces the concept of the psi degree of a vertex, defined through a combined contribution of the sum and the product of the degrees of its neighboring vertices, followed by an appropriate normalization. This construction is intended to capture more detailed information about the local bonding environment and collective interactions that influence π-electron delocalization in benzenoid systems. Based on this new vertex descriptor, three novel Zagreb psi indices are defined and investigated for the first time.

The chemical applicability of the proposed indices is evaluated through correlation analysis, regression modeling, and structural smoothness assessment, with particular emphasis on the π-electron energy of benzenoid hydrocarbons. The results are systematically compared with those of existing topological indices reported in the literature [21–25]. Finally, the advantages, limitations, and potential directions for future research are discussed, aiming to clarify the scope and predictive potential of Zagreb psi indices within QSPR studies.

## Basic definitions

The story of topological indices began in 1947, when Harold Wiener modelled the boiling points of alkanes using the index he defined [1]. The Wiener index is defined based on the concept of distance between vertices in a graph. Contrary to popular belief, the Zagreb index is not the first degree-based index. The Platt index, which was also defined in 1947 [2], was the first degree-based index. The Hosoya index took its place in the literature as the third topological index in 1971. For the interesting story of the Hosoya index, see reference [29]. And after that Randić and Zagreb indices were defined [4, 5].

Let $\:G$ be a graph, and $\:v$ be a vertex of this graph. This vertex’s degree, $\:deg\:v$, is the number of edges adjacent to it. We denote the set of vertices of a graph as $\:V\left(G\right)$, and the set of edges as $\:E\left(G\right).$ Table 1 below gives the definitions of degree-based topological indices found in the literature and used in this study. We selected the indices in Table 1 due to the smoothness analysis results provided by Kumar and Das in 2024 [3]. We will be able to compare these results with the Zagreb psi indices we define in this article in the fifth section of this study.

**Table 1:** Indices and their definitions

Name of index	Symbol	Formula	Reference
The first Zagreb	$\:{M}_{1}$	$\:{M}_{1}={\sum\:}_{v\in\:V\left(G\right)}{\left(deg\:v\right)}^{2}$	[4]
The second Zagreb	$\:{M}_{2}$	$\:{M}_{2}={\sum\:}_{uv\in\:E\left(G\right)}{deg}u\cdot\:{deg}v$	[4]
Randić	$\:R$	$\:R={\sum\:}_{uv\in\:E\left(G\right)}\frac{1}{\sqrt{{deg}u\cdot\:{deg}v}}$	[5]
Reciprocal Randić	$\:RR$	$\:RR=$ ${\sum\:}_{uv\in\:E\left(G\right)}\sqrt{{deg}u\cdot\:{deg}v}$	[6]
Sum-connectivity	$\:SCI$	$\:SCI=$ ${\sum\:}_{uv\in\:E\left(G\right)}\frac{1}{\sqrt{{deg}u+{deg}v}}$	[7]
Symmetric division deg	$\:SDD$	$\:SDD={\sum\:}_{uv\in\:E\left(G\right)}(\frac{{deg}u}{{deg}v}+\frac{{deg}v}{{deg}u})$	[8]
Harmonic	$\:H$	$\:H={\sum\:}_{uv\in\:E\left(G\right)}\frac{1}{\sqrt{{deg}u+{deg}v}}$	[9]
Inverse sum indeg	$\:ISI$	$\:ISI={\sum\:}_{uv\in\:E\left(G\right)}\frac{{deg}u\cdot\:{deg}v}{{deg}u+{deg}v}$	[8]
Atom-bond connectivity	$\:ABC$	$\:ABC=$ ${\sum\:}_{uv\in\:E\left(G\right)}\sqrt{\frac{{deg}u+{deg}v-2}{{deg}u\cdot\:{deg}v}}$	[10]
Augmented Zagreb index	$\:AZI$	$\:AZI=$ ${\sum\:}_{uv\in\:E\left(G\right)}{\left(\frac{{deg}u\cdot\:{deg}v}{{deg}u+{deg}v-2}\right)}^{3}$	[11]
The first hyper-Zagreb	$\:{HM}_{1}$	$\:{HM}_{1}=$ ${\sum\:}_{uv\in\:E\left(G\right)}{\left({deg}u+{deg}v\right)}^{2}$	[12]
The second hyper-Zagreb	$\:{HM}_{2}$	$\:{HM}_{2}=$ ${\sum\:}_{uv\in\:E\left(G\right)}{\left({deg}u\cdot\:{deg}v\right)}^{2}$	[13]
Geometric-arithmetic	$\:GA$	$\:GA={\sum\:}_{uv\in\:E\left(G\right)}\frac{2\sqrt{{deg}u\cdot\:{deg}v}}{{deg}u+{deg}v}$	[14]
The fourth geometric-arithmetic	$\:{GA}_{4}$	$\:{GA}_{4}={\sum\:}_{uv\in\:E\left(G\right)}\frac{2\sqrt{{\epsilon\:}_{u}{\epsilon\:}_{v}}}{{\epsilon\:}_{u}+{\epsilon\:}_{v}}$	[15]
Arithmetic-geometric index	$\:AG$	$\:AG={\sum\:}_{uv\in\:E\left(G\right)}\frac{{deg}u+{deg}v}{2\sqrt{{deg}u\cdot\:{deg}v}}$	[16]
Sombor	$\:SO$	$\:SO=$ ${\sum\:}_{uv\in\:E\left(G\right)}\sqrt{{\left(deg\:u\right)}^{2}+{\left(deg\:v\right)}^{2}}$	[17]
Modified Sombor	$\:{SO}^{m}$	$\:{SO}^{m}=$ ${\sum\:}_{uv\in\:E\left(G\right)}\frac{1}{\sqrt{{{deg}u}^{2}+{{deg}v}^{2}}}$	[18]
Nirmala	$\:N$	$\:N={\sum\:}_{uv\in\:E\left(G\right)}\sqrt{{deg}u+{deg}v}$	[19]
The first inverse Nirmala	$\:{IN}_{1}$	$\:{IN}_{1}=$ ${\sum\:}_{uv\in\:E\left(G\right)}\sqrt{\frac{1}{{deg}u}+\frac{1}{{deg}v}}$	[20]
The second inverse Nirmala	$\:{IN}_{2}$	$\:{IN}_{2}={\sum\:}_{uv\in\:E\left(G\right)}\frac{1}{\sqrt{\frac{1}{{deg}u}+\frac{1}{{deg}v}}}$	[20]

Now we can give the definition of the psi degree of a vertex and the definitions of three different Zagreb psi indices, which form the basis of this study.

To incorporate both local connectivity and collective neighborhood interactions into a single vertex-level quantity, we introduce the psi degree, which combines additive and multiplicative contributions of neighboring vertex degrees in a normalized form.

### Definition 2.1

Let $\:G$ be an $\:n$-vertex connected graph, and let $\:v$ be a vertex of $\:G$. The psi degree of the vertex $\:v$ is defined as,

$$\:\psi\:\left(v\right)=\frac{1}{\sqrt[n]{{S}_{v}+{M}_{v}}}$$

Here, $\:{S}_{v}$ is the sum of the degrees of all vertices neighboring *v*, and $\:{M}_{v}$ is the product of the degrees of all vertices neighboring *v*.

### Definition 2.2

The first Zagreb psi index of an $\:n$-vertex connected graph $\:G$ is defined as;

$$\:{M}_{1}{\Psi\:}\left(G\right)={\sum\:}_{v\in\:V\left(G\right)}{\left(\psi\:\left(v\right)\right)}^{2}$$

### Definition 2.3

The second Zagreb psi index of an $\:n$-vertex connected graph $\:G$ is defined as;

$$\:{M}_{2}{\Psi\:}\left(G\right)={\sum\:}_{uv\in\:E\left(G\right)}\psi\:\left(u\right)\cdot\:\psi\:\left(v\right)$$

### Definition 2.4

The third Zagreb psi index of an $\:n$-vertex connected graph $\:G$ is defined as;

$$\:{M}_{3}{\Psi\:}\left(G\right)={\sum\:}_{uv\in\:E\left(G\right)}\left(\psi\:\left(u\right)+\psi\:\left(v\right)\right)$$

Now, we give the values of Zagreb psi indices in simple graph classes such as path, cycle, star and complete graphs in Table 2.

**Table 2:** Zagreb psi indices values in basic graph classes

Index	Path (*P*_*n*_)	Cycle (C_*n*_)	Star (S_*n*_)	Complete (K_*n*_)
$\:{M}_{1}{\Psi\:}$	$\:2\times\:{16}^{-1/n}+2\times\:{25}^{-1/n}+$ $+\left(n-4\right)\times\:{64}^{-1/n}$	$\:n\times\:{64}^{-1/n}$	$\:{n}^{-2/n}+$ $+\left(n-1\right)\times\:{\left(4{\left(n-1\right)}^{2}\right)}^{-1/n}$	$\:n\times\:{\left({\left(n-1\right)}^{2}+{\left(n-1\right)}^{n-1}\right)}^{-2/n}$
$\:{M}_{2}{\Psi\:}$	$\:2\times\:{20}^{-1/n}+2\times\:{40}^{-1/n}+$ $+\left(n-5\right)\times\:{64}^{-1/n}$	$\:n\times\:{64}^{-1/n}$	$\:\left(n-1\right)\times$ ${\left(2{n}^{2}-2n\right)}^{-1/n}$	$\:\frac{n(n-1)}{2}\times\:{\left({\left(n-1\right)}^{2}+{\left(n-1\right)}^{n-1}\right)}^{-2/n}$
$\:{M}_{3}{\Psi\:}$	$\:2\times\:{4}^{-1/n}+4\times\:{5}^{-1/n}+$ $+2\left(n-4\right)\times\:{8}^{-1/n}$	$\:2n\times\:{8}^{-1/n}$	$\:\left(n-1\right)\times\:$ $\left({n}^{-1/n}+{\left(2n-2\right)}^{-1/n}\right)$	$\:n(n-1)\times\:{\left({\left(n-1\right)}^{2}+{\left(n-1\right)}^{n-1}\right)}^{-1/n}$

## Computational methods

All molecular graphs considered in this study were treated as simple, undirected, and connected graphs. Benzenoid hydrocarbons were represented by their corresponding hexagonal systems, where vertices denote carbon atoms and edges represent carbon–carbon bonds. Hydrogen atoms were omitted, following standard practice in chemical graph theory and QSPR studies [4, 21].

Vertex degrees were computed directly from the molecular graphs, and the psi degree of each vertex was calculated according to the definition introduced in this work. Based on these psi degrees, three Zagreb psi indices were evaluated for each benzenoid structure. For comparison purposes, the values of several well-established topological indices reported in the literature were also considered, including classical Zagreb-type indices, degree-based descriptors, and selected distance-based indices [6–8, 17–24].

The π-electron energy values of benzenoid hydrocarbons were taken from previously published sources and used as reference physicochemical data [4, 21–23]. To assess the chemical applicability of the proposed indices, Pearson’s correlation coefficients and linear regression models were employed. The regression parameters, including correlation coefficients and goodness-of-fit measures, were evaluated using standard least-squares procedures.

The statistical significance of the obtained correlation coefficients was further examined by computing the corresponding p-values. For all reported correlations between the Zagreb psi indices and the π-electron energy of benzenoid hydrocarbons, the p-values were found to be well below 0.001, indicating a statistically highly significant relationship. Given the relatively small size of the dataset and the linear nature of the regression models considered in this study, additional cross-validation procedures were not applied, as the primary objective is the comparative evaluation and chemical applicability of newly introduced topological indices rather than predictive generalization.

In addition to correlation analysis, the structural behavior of the Zagreb psi indices was examined through structure sensitivity (SS) and abruptness (Abr) analyses. These analyses were conducted following established methodologies in the literature, which assess how sensitively a topological index responds to local structural modifications in molecular graphs [3, 26–28]. Such evaluations are essential for determining whether a descriptor provides chemically meaningful discrimination between closely related structures.

All calculations were carried out using standard mathematical procedures implemented in a symbolic and numerical computation environment. No fitting parameters were introduced in the definition of the Zagreb psi indices, ensuring that the proposed descriptors remain purely structure-based and free from empirical adjustments.

All benzenoid hydrocarbons considered in this study are standard structures that have been extensively investigated in the literature. Their molecular graphs follow the conventional hexagonal representations commonly used in π-electron energy studies, and the corresponding π-electron energy values were taken from well-established reference sources. Therefore, no additional atom numbering scheme is required for reproducibility.

## Zagreb psi indices for benzenes: results and discussion

This section presents and discusses the correlation, comparative performance, and structural behavior of the Zagreb psi indices for benzenoid hydrocarbons. In particular, it reports the highest correlation coefficients obtained to date between degree-based topological indices and the π-electron energy of benzenes. First, the values of the newly defined Zagreb psi indices for benzenoid hydrocarbons are presented. The obtained results demonstrate that the third Zagreb psi index exhibits the highest correlation with the π-electron energy of benzenes among all degree-based molecular topological indices reported so far. Accordingly, a mathematical model for the π-electron energy of benzenes based on the Zagreb psi indices is proposed. For comparison purposes, Table 3 summarizes the highest correlation coefficients reported in previous studies on the π-electron energy of benzenoid hydrocarbons.

Despite the extensive literature on degree-based and distance-based topological indices, most existing descriptors rely exclusively on either additive or multiplicative information of vertex degrees, which may limit their ability to capture local structural interactions in conjugated systems. The present study addresses this gap by introducing the psi degree, a unified vertex descriptor that simultaneously incorporates both additive and multiplicative contributions of neighboring degrees in a mathematically simple and chemically interpretable form. As demonstrated by the comparative correlation analysis, the resulting Zagreb psi indices, particularly the third Zagreb psi index, outperform all previously reported degree-based descriptors for modeling the π-electron energy of benzenoid hydrocarbons. This improved performance, combined with favorable smoothness and structural sensitivity properties, highlights the advantage of the proposed indices as robust and chemically meaningful alternatives within the family of degree-based topological descriptors.

To further clarify the relationship between the proposed Zagreb psi indices and other well-known molecular descriptors, a direct comparison with representative degree-based and distance-based topological indices reported in the literature was carried out. As shown in Tables 3 and 4, classical degree-based indices such as the Zagreb and Randić indices exhibit strong but comparatively lower correlations with the π-electron energy of benzenoid hydrocarbons. Distance-based descriptors, particularly Harary energy, achieve slightly higher correlation values; however, these indices rely on global distance information rather than purely local degree characteristics. In this context, the third Zagreb psi index demonstrates that incorporating combined local information from neighboring vertex degrees allows degree-based descriptors to approach the predictive performance of distance-based measures, while preserving structural interpretability and computational simplicity.

**Table 3:** π-electron energy of benzenes with highest correlation coefficients

Topological index	Symbol	Correlation coefficient	Reference
Randić index (degree-based)	$\:R$	0.9993	[21]
Fourth geometric–arithmetic index (distance-based)	$\:{GA}_{4}$	0.9991	[22]
Harary Energy (distance-spectral)	$\:{E}_{H}$	0.99964	[23]
Elliptic Sombor index (degree-based)	*ESO*	0.995	[24]

For clarity, a concise comparative ranking of the best-performing topological indices is provided in Table 4 to directly illustrate the relative position of the proposed Zagreb psi index.

**Table 4:** Comparative performance of selected topological indices for modeling the π-electron energy of benzenoid hydrocarbons, ranked according to decreasing correlation coefficients

Rank	Topological index	Symbol	Correlation coefficient
1	Randić index (degree-based)	$\:R$	0.9993
2	Fourth geometric–arithmetic index (distance-based)	$\:{GA}_{4}$	0.9991
3	Harary Energy (distance-spectral)	$\:{E}_{H}$	0.99964
*4*	Elliptic Sombor index (degree-based)	*ESO*	0.995
5	Third Zagreb psi index	M_3_$\Psi$	0.99948

Table 4 includes selected representative degree-based and distance-spectral indices that exhibit the highest reported correlations with the π-electron energy of benzenoid hydrocarbons. All correlation values reported in this table are reproduced from the corresponding detailed correlation tables presented in this study (see Table 3) and from the referenced literature. The complete set of correlation results is provided in Table 3. This table is intended to provide a direct and transparent ranking of the best-performing descriptors, clearly highlighting the relative position of the third Zagreb psi index (M_3_Ψ) among well-established degree-based and distance-based topological indices

Table 4 provides a direct ranking of the best-performing topological indices for modeling the π-electron energy of benzenoid hydrocarbons. As seen, the third Zagreb psi index (M_3_${\Psi}$) achieves a correlation coefficient of 0.99948, ranking immediately after Harary Energy and outperforming all other degree-based descriptors reported so far. This result highlights the strong predictive potential of the proposed index while placing its performance in a clear comparative context.

Table 5 shows the calculated values of Zagreb psi indices in benzenes.

**Table 5:** Zagreb psi indices of benzenes

Benzenes	$\:\pi\:-$electron energy of benzenes	The first Zagreb Psi index	The second Zagreb Psi index	The third Zagreb psi index
Benzene	8.000	3.000	3.000	8.485
Naphthalene	13.683	6.225	6.775	17.262
Phenanthrene	19.448	9.801	11.080	26.623
Anthracene	19.314	9.797	11.107	26.657
Chrysene	25.192	13.531	15.638	36.239
Benzo[a]anthracene	25.101	13.528	15.663	36.267
Triphenylene	25.275	13.536	15.619	36.214
Tetracene	25.188	13.526	15.686	36.296
Benzo[a]pyrene	28.222	15.316	18.221	41.817
Benzo[e]pyrene	28.336	15.285	18.167	41.755
Perylene	28.245	15.319	18.200	41.793
Anthanthrene	31.253	17.155	20.890	47.491
Benzo[g, h,i]perylene	31.425	17.133	20.846	47.441
Dibenz[a, c]nthracene	30.942	17.348	20.339	45.986
Dibenz[a, h]anthracene	30.881	17.343	20.357	46.007
Dibenz[a, j]anthracene	30.880	17.344	20.356	46.008
Picene	30.943	17.344	20.337	45.984
Coronene	34.572	18.972	23.540	53.144
Dibenzo[a, h]pyrene	33.928	19.174	23.000	51.645
Dibenzo[a, i]pyrene	33.954	19.174	23.000	51.645
Dibenzo[a, l]pyrene	34.031	19.175	22.982	51.624
Pyrene	22.506	11.619	13.685	32.249

Table 6 shows the correlation coefficients between the π-electron energy of benzenes and the Zagreb psi indices.

**Table 6:** The correlation coefficients between the π-electron energy of benzenes and the Zagreb psi indices

Name of the index	Symbol	Correlation coefficient
The first Zagreb Psi index	$\:{M}_{1}{\Psi\:}$ (G)	0.99897
The second Zagreb Psi index	$\:{M}_{2}{\Psi\:}$ (G)	0.99913
The third Zagreb Psi index	$\:{M}_{3}{\Psi\:}$ (G)	0.99948

**Fig. 1:**
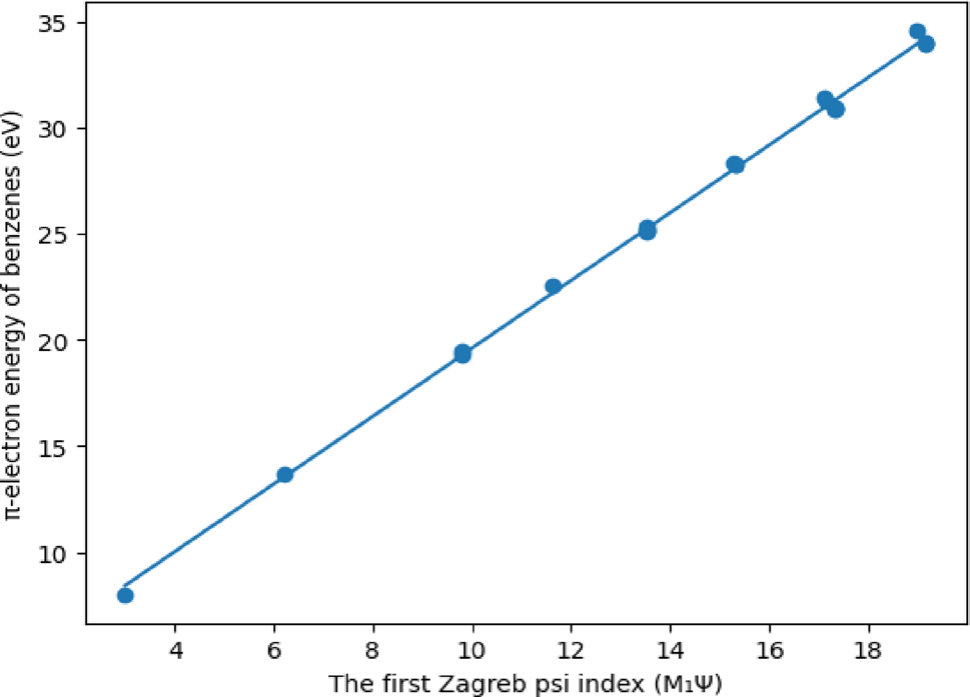
Linear regression between the π-electron energy of benzenoid hydrocarbons and the first Zagreb psi index (M_1_${\Psi}$)

**Fig. 2:**
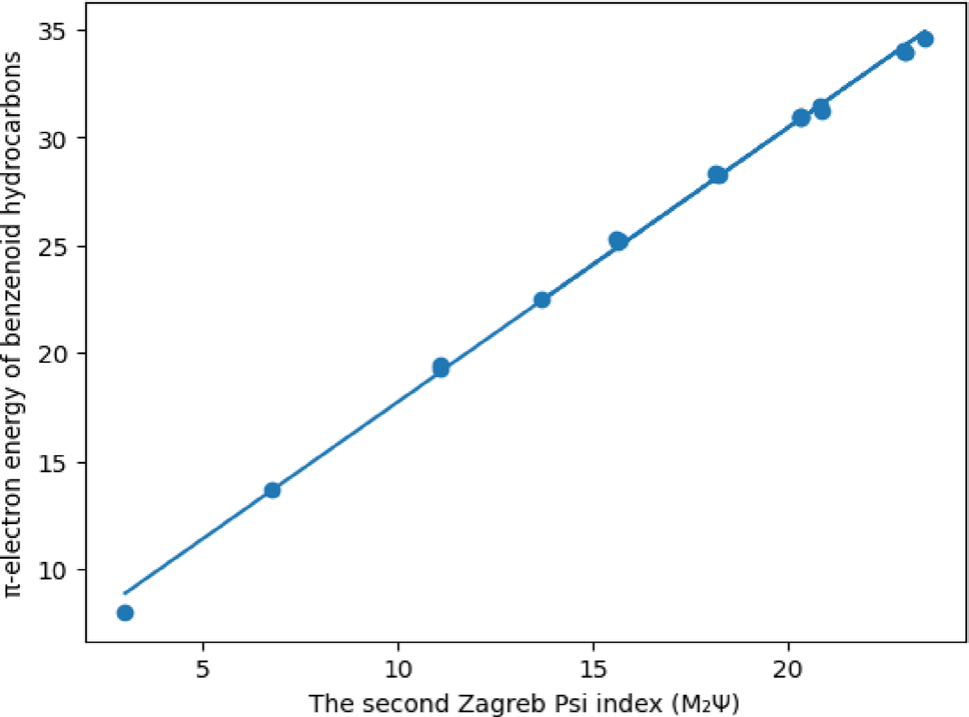
Linear regression between the π-electron energy of benzenoid hydrocarbons and the second Zagreb psi index (M_2_${\Psi}$)

**Fig. 3:**
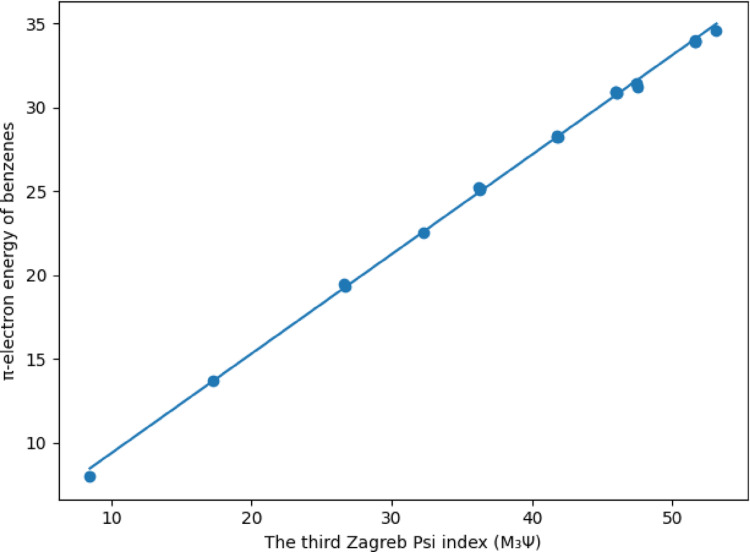
Linear regression modeling of π-electron energy of benzenoid hydrocarbons using the third Zagreb psi index ($\:{M}_{3}{\Psi\:}$)

As can be seen from Table 6, the correlation coefficients between the three new indices and the $\:\boldsymbol{\pi\:}-$electron energy levels of benzenes are greater than 0,99. The highest correlation coefficient in the literature belongs to the Harary Energy index with 0.99964. As can be seen, the third Zagreb Psi index with a correlation value of 0.99948 gave the second highest correlation value so far. Additionally, this ratio shows the highest rate among degree-based topological indices. Therefore, it is seen that the first condition required to define a new index presented in this article is met. Linear regression modelling of the $\:\boldsymbol{\pi\:}-$electron energy levels of benzenes will be given in Figs. 1, 2 and 3; Tables 6, 7, 8 and 9.

The technical details of the graph given in Fig. 1 are given below in Table 7.

**Table 7:** Details of linear regression model of π-electron energy levels of benzenes via the first Zagreb psi index

Parameter	Value
Intercept	3.65195
Slope	1.59359
Pearson’s R	0.99897
R-Square (COD)	0.99794
Adj. R-Square	0.99784

The technical details of the graph given in Fig. 2 are given below in Table 8.

**Table 8:** Details of linear regression model of π-electron energy levels of benzenes via the second Zagreb psi index

Parameter	Value
Intercept	5.06231
Slope	1.59359
Pearson’s R	0.99913
R-Square (COD)	0.99826
Adj. R-Square	0.99817

The technical details of the graph given in Fig. 3 are given below in Table 9.

**Table 9:** Details of linear regression model of π-electron energy levels of benzenes via the third Zagreb psi index

Parameter	Value
Intercept	3.45211
Slope	0.59332
Pearson’s R	0.99948
R-Square (COD)	0.99896
Adj. R-Square	0.9989

## Relations with other indices

This section provides correlation coefficients between the Zagreb psi indices and well-known degree-based topological indices such as; Randić (R), Atom-Bond Connectivity (ABC), Augmented Zagreb (AZI), Geometric-Arithmetic (GA), the first and second Zagreb (M_1_, M_2_), Sombor (S) topological indices. The values taken from the references [21] and [25].

**Table 10:** Well-known degree based topological indices of benzenes

Benzenes	*R*	ABC	AZI	GA	M_1_	M_2_	SO
Benzene	3.000	4.2426	48	6	24	24	16.9706
Naphthalene	4.966	7.7377	91.3906	10.9192	50	57	35.6354
Phenanthrene	6.950	11.1924	138.1719	15.8788	76	91	54.1602
Anthracene	6.933	11.2328	134.7813	15.8384	76	90	54.3003
Chrysene	8.933	14.647	184.9531	20.8384	102	125	72.785
Benzo[a]anthracene	8.916	14.6875	181.5625	20.798	102	124	72.8251
Triphenylene	8.950	14.6066	188.3438	20.8788	102	126	72.545
Tetracene	8.899	14.7279	178.1719	20.7576	102	123	72.9651
Benzo[a]pyrene	9.916	16.647	219.125	23.8384	120	152	85.413
Benzo[e]pyrene	9.933	16.647	219.125	23.798	120	151	85.553
Perylene	9.933	16.647	219.125	23.8384	120	152	85.413
Anthanthrene	10.899	18.7279	246.5156	26.7576	138	177	98.4209
Benzo[g, h,i] perylene	10.916	18.6875	249.9063	26.798	138	178	98.2809
Dibenz[a, c]anthracene	10.916	18.1017	231.7344	25.798	128	159	91.2098
Dibenz[a, h]anthracene	10.899	18.1421	228.3438	25.7576	128	158	91.3499
Dibenz[a, j]anthracene	10.899	18.1421	228.3438	25.7576	128	158	91.3499
Picene	10.915	18.1017	231.7344	25.798	128	159	91.2098
Coronene	11.899	20.7279	280.6875	29.7576	156	204	111.1489
Dibenzo[a, h]pyrene	11.582	20.1421	262.5156	28.7576	146	185	104.0778
Dibenzo[a, i]pyrene	11.566	20.1421	262.5156	28.7576	146	185	104.0778
Dibenzo[a, l]pyrene	11.491	20.1017	265.9063	28.798	146	186	103.9378
Pyrene	11.915	13.2328	168.9531	18.8384	94	117	67.0282

Table 10 shows the correlations between the Randić (R), the Randić (R), Atom-Bond Connectivity (ABC), Augmented Zagreb (AZI), Geometric-Arithmetic (GA), the first and second Zagreb (M_1_, M_2_), Sombor (SO) topological indices and the newly defined Zagreb psi indices.

**Table 11:** The correlation coefficients between the well-known topological indices and the Zagreb psi indices

Zagreb Psi index	*R*	ABC	AZI	GA	M_1_	M_2_	SO
The first Zagreb Psi index	0.92728	0.99774	0.99059	0.99711	0.99219	0.98516	0.99233
The second Zagreb Psi index	0.92895	0.99927	0.99593	0.99925	0.99713	0.99275	0.99719
The third Zagreb Psi index	0.93161	0.99981	0.99673	0.99981	0.99802	0.99385	0.99808

As can be seen from Table 11, the correlation coefficients between the Zagreb psi indices, and the Randić (R), Atom-Bond Connectivity (ABC), Augmented Zagreb (AZI), Geometric-Arithmetic (GA), the first and second Zagreb (M1, M2), Sombor (SO) topological indices are greater than 0.99. This indicates a very strong relationship. The lowest correlation between Zagreb psi indices and the Randić index is 0.92728, indicating a relatively good relationship.

## Smoothness analysis

In this section, we explore the smoothness characteristics of specific Zagreb psi topological indices and perform a comparative assessment against the established results related to various prominent topological indices. Two novel graph structural metrics, specifically structure sensitivity (abbreviated as SS) and abruptness (represented as Abr), were introduced in [26] to evaluate the smoothness of a molecular descriptor. Recent scholarly works have examined the structure sensitivity (SS) of eigenvalue-based topological indices and the smoothness of graph energy within chemical graphs in the publications [27] and [28], respectively. See the reference 26 for the algorithm designed to compute the SS and Abr of a topological index pertaining to a specified category of connected graphs [26]. Kumar and Das performed the smoothness analysis of the fifteen degree-based topological indices which given in Table 1 for all tree graphs between 4 vertices to 10 vertices by using the algorithm designed to compute the SS and Abr of a topological index.

The structural sensitivity (SS) and abruptness (Abr) analyses provide additional insight into the behavior of the proposed Zagreb psi indices beyond their predictive accuracy. In chemical graph theory, an effective molecular descriptor should respond to local structural modifications in a controlled and continuous manner, avoiding both excessive insensitivity and abrupt fluctuations. The obtained SS and Abr values indicate that the Zagreb psi indices, particularly the third Zagreb psi index, exhibit moderate and stable structural responsiveness. This behavior suggests that the proposed indices are capable of distinguishing closely related benzenoid structures while preserving smooth variation across small structural changes. Such a balance between sensitivity and smoothness is widely regarded as a desirable property for chemically meaningful and interpretable topological descriptors, supporting the robustness and reliability of the Zagreb psi indices for QSPR applications.

Using the same algorithm, the structure sensitivity (SS) and abruptness (Abr) analysis results of Zagreb psi indices are calculated for tree graphs from four to nine vertices and are given in Table 12.

**Table 12:** Structure sensitivity (SS) and abruptness (Abr) analysis results of Zagreb psi indices on tree graphs

Zagreb Psi Indices	n = 4		n = 5		n = 6		n = 7		n = 8		n = 9	
	SS	Abr	SS	Abr	SS	Abr	SS	Abr	SS	Abr	SS	Abr
M_1_${\Psi\:}$	0.0936	0.0936	0.0759	0.0826	0.0412	0.0586	0.0304	0.0495	0.0226	0.0369	0.0203	0.0413
M_2_${\Psi\:}$	0.0277	0.0277	0.0359	0.0393	0.0329	0.0553	0.0232	0.0414	0.0197	0.036	0.0176	0.0375
M_3_${\Psi\:}$	0.0128	0.0128	0.0175	0.0191	0.0155	0.027	0.0114	0.0203	0.0097	0.0178	0.0087	0.0183

For the efficacy of a topological index, it is essential that the SS value be maximized while concurrently minimizing the Abr value. In the nine-vertex tree graphs, when we compare the results in Table 12 with the results in Table 1 of the third reference and Table 3 of the twenty-sixth reference, it is observed that the SS value of the Zagreb Psi indices is only greater than that of the first inverse Nirmala index. Again, in parallel with this result, it is observed that the Abr value of the Zagreb Psi indices is only greater than that of the first inverse Nirmala index. Therefore, the fact that the Abr values ​​are close to the minimum can be seen as an advantage for the Zagreb psi indices. However, the fact that the SS values are close to the maximum can be interpreted as a disadvantage for the Zagreb psi indices.

## Conclusion

In this study, a new vertex descriptor, referred to as the psi degree, was introduced by combining additive and multiplicative contributions of neighboring vertex degrees into a unified formulation. Based on this concept, three Zagreb-type psi indices were defined and systematically analyzed within a QSPR framework.

The correlation and regression analyses demonstrated that the third Zagreb psi index (M_3_Ψ) exhibits an exceptionally strong relationship with the π-electron energy of benzenoid hydrocarbons, achieving a correlation coefficient of 0.99948. This value places M_3_Ψ immediately after Harary energy and establishes it as the most effective degree-based topological index reported so far for modeling π-electron energy of benzenes.

In addition to predictive performance, the structural behavior of the proposed indices was examined through structural sensitivity and smoothness analyses. The results indicate that while the Zagreb psi indices display meaningful structural discrimination, their sensitivity remains at a moderate level compared to some highly structure-sensitive descriptors. This balance between predictive accuracy and structural smoothness suggests that the proposed indices provide chemically interpretable information without excessive sensitivity to minor structural variations.

The present study is limited to benzenoid hydrocarbons and linear regression models. Future work may extend the application of Zagreb psi indices to other molecular classes, explore their performance in modeling different physicochemical properties, and investigate nonlinear or multivariate modeling approaches. Overall, the results indicate that Zagreb psi indices constitute promising and chemically meaningful tools for QSPR studies.

## Data Availability

No new data were generated or analyzed in this study.
